# Inhalable Combination Powder Formulations for Treating Latent and Multidrug-Resistant Tuberculosis: Formulation and In Vitro Characterization

**DOI:** 10.3390/pharmaceutics15092354

**Published:** 2023-09-20

**Authors:** Basanth Babu Eedara, Claire Fan, Shubhra Sinha, Prakash Khadka, Shyamal C. Das

**Affiliations:** 1 School of Pharmacy, University of Otago, Dunedin 9054, New Zealand; 2 Transpire Bio Inc., 2945 W Corporate Lakes Blvd Suite A, Weston, FL 33331, USA; 3 Department of Physiology, Heart Otago, School of Biomedical Sciences, University of Otago, 270 Great King Street, P.O. Box 913, Dunedin 9054, New Zealand

**Keywords:** tuberculosis, pyrazinamide, PA-824, moxifloxacin, inhalation, aerosolization, spray drying, dry powder inhaler

## Abstract

Tuberculosis (TB) is an infectious disease resulting in millions of deaths annually worldwide. TB treatment is challenging due to a huge number of global latent infections and due to multidrug-resistant forms of TB. Inhaled administration of anti-TB drugs using dry powder inhalers has various advantages over oral administration due to its direct drug delivery and minimization of systemic side effects. Pretomanid (PA-824, PA) is a relatively new drug with potent activity against both active and latent forms of *Mycobacterium tuberculosis* (Mtb). It is also known for its synergistic effects in combination with pyrazinamide (PYR) and moxifloxacin (MOX). Fixed-dose combination powder formulations of either PYR and PA or PYR and MOX were prepared for inhaled delivery to the deep lung regions where the Mtb habitats were located. Powder formulations were prepared by spray drying using L-leucine as the aerosolization enhancer and were characterized by their particle size, morphology and solid-state properties. In vitro aerosolization behaviour was studied using a Next Generation Impactor, and stability was assessed after storage at room temperature and 30% relative humidity for three months. Spray drying with L-leucine resulted in spherical dimpled particles, 1.9 and 2.4 µm in size for PYR-PA and PYR-MOX combinations, respectively. The powder formulations had an emitted dose of >83% and a fine particle fraction of >65%. PA and MOX showed better stability in the combination powders compared to PYR. Combination powder formulations with high aerosolization efficiency for direct delivery to the lungs were developed in this study for use in the treatment of latent and multidrug-resistant TB infections.

## 1. Introduction

Tuberculosis (TB) is a disease mainly of the lungs caused by *Mycobacterium tuberculosis* (Mtb), and it was one of the leading causes of death worldwide due to a single microorganism until 2021 [1]. In 2021, TB was the second leading infectious killer behind coronavirus disease 2019 (COVID-19) [1,2]. Although predominantly localized in the lungs as pulmonary TB, it can also affect sites other than the lungs, known as extra-pulmonary TB in 10 to 42% of TB cases [3]. Around 10.6 million new TB cases and 1.4 million TB-related deaths were reported by the World Health Organization (WHO) in 2021 [1]. Asymptomatic infection with Mtb or latent TB cases is estimated to include a quarter of the world’s population, corresponding to approximately two billion people [1,4,5]. Although only about 10% of those latently infected eventually develop active disease, these infected individuals serve as the reservoir for the Mtb, which poses challenges for detection, prevention, treatment and control of the disease [6]. Growing drug resistance and co-infection with human immunodeficiency virus (HIV) pose additional obstacles to developing a successful TB treatment regimen [7,8,9].

The currently recommended treatment regimen for drug-susceptible TB includes oral treatment with first-line agents for a two-month ‘intensive phase’, followed by a four-month ‘continuation phase’. In the initial intensive phase, the drugs rifampicin, isoniazid, pyrazinamide and ethambutol are used in combination for treatment [7]. In spite of a cure rate exceeding 95% in trial conditions over its 25+ year widespread usage [3], this four-drug treatment regimen for drug-sensitive TB is limited by the lengthy treatment duration, drug side effects and, perhaps of even greater concern, developing drug resistance [8].

TB patients who do not respond to the first-line treatment are classified as having drug-resistant TB, which can be either multidrug-resistant TB (MDR-TB) or extensively drug-resistant TB (XDR-TB). In MDR-TB, the mycobacteria are resistant to at least isoniazid and rifampicin, the two potent first-line anti-TB agents. In some cases, more severe drug resistance can develop as XDR-TB, which is an aggravation of MDR-TB with further resistance to the fluoroquinolones and at least one of the injectable second-line drugs—capreomycin, kanamycin and amikacin. Due to the huge economic and public health burden of drug-resistant TB, there is an urgent need for new and potent treatment strategies that efficiently kill the mycobacteria and shorten the duration of treatment [9]. MDR-TB-targeting drugs are considered second-line because of their greater risk of intolerance, reduced potency, and more adverse side effect profile than the standard first-line regimen for drug-susceptible TB [10]. The WHO consolidated guidelines for drug-resistant TB treatment recommend several regimens lasting six months or longer for the treatment of MDR and rifampicin-resistant (RR) TB, with varying levels of certainty of evidence based on clinical trials [7]. One of the new recommendations includes the use of a 6-month treatment regimen composed of bedaquiline, pretomanid (PA-824, PA), linezolid (600 mg) and moxifloxacin (MOX) instead of the 9-month or longer regimens in MDR/RR-TB patients. Similarly, a shorter, 9-month all-oral regimen rather than longer regimens is recommended in patients with MDR/RR-TB and in whom resistance to fluoroquinolones has been excluded.

**Figure 1:**
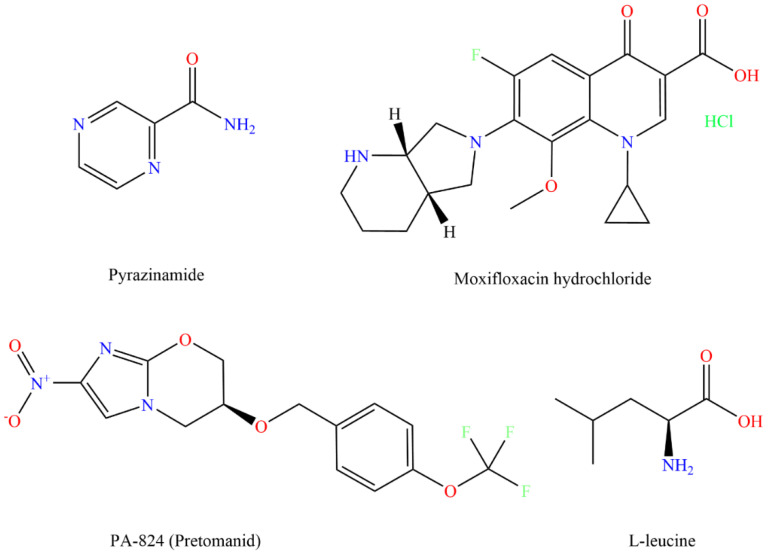
Chemical structures (drawn using Chem Draw 19.1, PerkinElmer, Waltham, MA, USA) of anti-TB drugs, pyrazinamide, moxifloxacin hydrochloride, PA-824 and excipient and L-leucine.

Currently, the use of fixed-dose combination tablets is recommended over separate drug formulations in the treatment of patients with drug-susceptible TB [7]. A fixed-dose combination in TB treatment is an approach that allows simultaneous administration of multiple drugs in a combined form and has the potential to improve patient compliance and reduce medication errors [11]. In this study, fixed-dose combination inhalable dry powder formulations containing either PYR and PA or PYR and MOX have been investigated. PYR (Figure 1) is a first-line drug for drug-sensitive TB, which is also used in MDR-TB to shorten the therapy. MOX (Figure 1), a fluoroquinolone antibiotic, is a second-line anti-TB agent with bactericidal activity against Mtb and has shown promising results in shortening TB treatment time and reducing toxicity [12]. PA (Figure 1), also known as pretomanid, is a novel anti-TB agent that has bactericidal activity against both actively replicating and hypoxic non-replicating MTB strains and thus has the potential to treat MDR-TB [13]. In vitro studies and murine models have demonstrated its substantial activity against a variety of drug-sensitive as well as drug-resistant Mtb isolates and non-replicating (persistent) bacteria [14,15,16]. The fixed-dose combination approach for anti-TB drugs has the potential to benefit patients with drug sensitive or MDR-TB and shorten the treatment duration [17].

Inhaled delivery of anti-TB drugs as dry powder inhalers (DPIs) is a safe, reproducible, non-invasive and convenient approach to delivering high concentrations of drugs to the lungs as well as systemic circulation. This route of drug administration can also achieve high bioavailability due to avoidance of the first-pass metabolism, can reduce the side effects that result from excessive systemic exposure to the drugs and can prevent the potential drug resistance build-up [18,19,20]. DPI is advantageous over nebulizers and metered dose inhalers because they are propellant-free, portable, easy to use and low-cost devices, and the formulations have improved stability in a dry powder form [21]. Moreover, they are suitable for high-dose drug delivery, which is a prime requirement for antibiotic administration by inhalation in TB treatment. In the present study, combination drug powders were prepared by spray drying and using L-leucine (LEU, Figure 1) as an aerosolization enhancer to obtain a powder with high aerosolization potential for deep lung delivery.

## 2. Materials and Methods

### 2.1. Materials

The materials used in this study include the following: pyrazinamide (purity: 99.7%, Amsal Chem. Pvt. Ltd., Gujarat, India); PA-824 (99% purity, Carbosynth Ltd., Berkshire, UK); moxifloxacin hydrochloride (>98% purity, Leader Biochemical Group Xi’an Leader Biochemical Engineering Co., Ltd., Xi’an, China); L-leucine (98% purity, Hangzhou Dayangchem Co., Ltd., Hangzhou, China); silicone oil (P. Code 378321; viscosity 10 cSt, Sigma-Aldrich, St. Louis, MI, USA); and size-3 hard gelatine PEG capsules (kindly donated by Qualicaps, Osaka, Japan). All other chemicals and solvents used were of high-performance liquid chromatography (HPLC) grade and purchased from Merck, Germany. Freshly collected ultrapure water (Millipore^®^ Milli-Q^®^ Reagent Water System (Merck Millipore, Burlington, MA, USA) filtered through 0.45 μm membrane filter was used throughout the study.

### 2.2. Preparation of Spray-Dried Powder Formulations and Estimation of Percentage Yield

Two powder formulations were prepared with a fixed-dose combination of either PYR and PA with LEU or PYR and MOX with LEU. Feed solutions for the PYR-PA-LEU formulation were prepared by dissolving supplied PYR, PA and LEU at 71.1, 8.9 and 20% *w*/*w*, respectively, in ethanol and water (70:30% *v*/*v*), and those for PYR-MOX-LEU formulation were prepared by dissolving supplied PYR, MOX and LEU at 64, 16 and 20% *w*/*w*, respectively, in ethanol and water (70:30% *v*/*v*). The mass ratio of PYR:PA was 8:1, which was selected based on the dose used in the phase II study conducted by Dawson et al. in 2015 [22]. For the PYR-PA-LEU formulation, the feed solution was prepared by dissolving 355.5 mg PYR and 44.5 mg PA in 70 mL of pure ethanol, separately dissolving 100 mg LEU in 30 mL of filtered ultrapure water before mixing together to form a feed solution of 0.5% *w*/*v* final concentration and sonicated for 30 min prior to spray drying. Similarly, in the case of PYR-MOX-LEU formulation, the feed solution of 0.5% *w*/*v* final concentration was obtained by mixing the solutions containing 320 mg PYR in 70 mL ethanol and those containing 80 mg MOX and 100 mg LEU in 30 mL ultrapure water.

A BUCHI B-290 Mini Spray Dryer (BÜCHI Labortechnik AG, Flawil, Switzerland) with a high-performance cyclone connected to the B-295 Inert Loop in a closed mode was utilized to prepare spray-dried powder formulations. High-purity dry nitrogen gas was used as the atomizing drying gas. The stainless-steel inner nozzle tip diameter was 0.7 mm. The conditions for spray drying included an inlet temperature of 70 °C, 50% aspirator control, a feed flow rate of 2 mL/min and a spray flow rate of 819 L/h (height 65 mm). Under these conditions, the outlet temperature varied between 41 and 46 °C. The spray-dried powders were then collected and stored in screw-capped glass vials. These glass vials were protected from light and stored in a desiccator at room temperature (22 ± 3 °C) and at a relative humidity of 30 ± 2% until analysis.

The percentage yield (% yield) of the prepared powders was estimated by dividing the collected powder weight by the total weight of the solids used for preparation of feed solution and expressed as a percentage.

Physical mixtures of PYR-PA-LEU (71.1:8.9:20% *w*/*w*/*w*) and PYR-MOX-LEU (64:16:20% *w*/*w*/*w*) were prepared by simple mixing of accurately weighed quantities of respective drugs and LEU using a spatula for 5 min.

### 2.3. Drug Content Estimation

For determination of active ingredients (PYR, MOX and PA) in the spray-dried powder formulations, 5 mg of each powder sample (n = 3) was dissolved in 100 mL of 20:80% *v*/*v* water: methanol mixture in a volumetric flask, followed by shaking at room temperature in an orbital mixer incubator (Ratek Instruments Pty Ltd., Boronia, Australia) at a speed of 200 rpm for 30 min. The mixtures were then sonicated using a bath sonicator (Misonix Inc., Washington, DC, USA) for 15 min and agitated by a vortex mixer to fully dissolve the powder. The samples were then filtered through a 0.22 µm membrane filter, and the concentration of each active ingredient in the samples was measured using HPLC analysis.

### 2.4. Surface Morphology by Scanning Electron Microscopy (SEM) 

A field emission scanning electron microscope (JEOL 6700F, JEOL Ltd., Tokyo, Japan) was used to examine the surface morphology of powder samples. Each sample was mounted on a metal stub with a double-sided adhesive carbon tape by light dusting method. Prior to SEM imaging, the powder samples were coated with a thin layer of gold alloy (10 nm) in vacuum using the sputter Emitech K575X high-resolution sputter coater (EM Technologies Ltd., London, UK). The SEM micrographs were then obtained at various magnifications (×100, ×5000 and ×25,000) with an accelerating voltage of 5 kV.

### 2.5. Particle Size Determination from SEM Images

The geometric mean particle diameter of the spray-dried powder was determined from the SEM images using ImageJ 1.48 software (Image Processing and Analysis in Java; software available from https://imagej.net/ij/index.html accessed on 10 July 2023). Representative SEM images of each powder sample at various magnifications were analysed by measuring the diameter of a minimum of 300 particles.

### 2.6. Differential Scanning Calorimetry (DSC)

Thermal behaviour of the supplied materials and spray-dried powder formulations was characterized by DSC Q1000 (TA instruments, New Castle, DE, USA) calibrated with indium (calibration standard, purity > 99.9%). Approximately 5 mg of the powder sample was weighed into a standard flat-bottomed aluminium pan (TA instruments), and the exact weight of each sample was recorded. The pan loaded with sample was then hermetically sealed with the standard aluminium lid. All DSC samples were heated from 30 °C to 300 °C at a heating scan rate of 5 °C/min with nitrogen gas purge rate of 50 mL/min. For blank measurement and comparison, an empty aluminium pan pressed similarly to the sample pans was used.

### 2.7. Thermogravimetric Analysis (TGA)

Thermogravimetric analysis of the supplied materials and spray-dried powder formulations was performed through use of a TGA Q50 Thermogravimetric Analyser (TA instruments, USA). The platinum sample pan (TA instruments, USA) was loaded with approximately 5–10 mg of the powder sample and heated under a nitrogen purge (40 mL/min) from 30 °C to 300 °C at a rate of 5 °C/min. The percentage weight loss during the heating process was analysed using Universal Analysis 2000 software v. 4.1D (TA instruments, USA).

### 2.8. Hot-stage Microscopy

Thermo-microscopic changes of the supplied material and spray-dried powder formulations were recorded using a phase-contrast light microscope (Nikon Optiphot PFX, Tokyo, Japan) equipped with a polarizer, as well as a hot stage (Mettler Toledo FP82HT, Visp, Switzerland) with a Mettler Toledo FP90 central processor. A microscopic glass slide with a coverslip containing the sample was heated from 30 °C to 300 °C at a rate of 5 °C/min. The heating program was controlled using Image Pro Plus Software Version 2.7 (TWAIN interface, SDK), and images were digitally captured via an OPTIKAM PRO5 digital camera (OPTIKA SRL, Ponteranica, Italy).

### 2.9. X-ray Powder Diffraction (XRPD) Analysis

To investigate the crystallinity of the powder samples, analysis was carried out using an X-ray diffractometer (PANalytical X’Pert PRO MPD PW3040/60 XRD, Malvern Panalytical Ltd., Almelo, The Netherlands) equipped with a rapid real-time multistrip X’Celerator detector. Each sample was prepared by loading and compressing the powder in a flat plate geometry. Then, the sample was placed in the diffractometer for scanning over an angular range of 5–35° (2θ). Under ambient room conditions, the Cu Kα radiation operated at a generator power of 40 kV and current of 30 mA. The results were analysed by the PANalytical High Score software (version 4.0). 

### 2.10. Attenuated Total Reflectance—Fourier-Transform Infrared (ATR-FTIR) Spectroscopy

A Varian 3100 FTIR (Excalibur series; Varian Inc., Palo Alto, CA, USA) equipped with an attenuated total reflectance accessory (GladiATR^TM^, PIKE Technologies, Madison, WI, USA) was used to obtain ATR-FTIR spectra of the supplied materials and spray-dried powder formulations. The experimental procedure involved sample placement onto the ATR diamond crystal, followed by scanning (64 scans at 4 cm^−1^ resolution) over the wave number range of 4000–400 cm^−1^.

### 2.11. In Vitro Aerosol Dispersion Performance Using Next Generation Impactor (NGI)

The aerosol dispersion performance of spray-dried powder formulations was assessed utilizing a Next Generation Impactor (NGI; Copley Scientific, Nottingham, UK) equipped with a Copley HCP5 vacuum pump and a Copley TPK 2000 critical flow controller. To prevent particle bouncing, all stainless-steel NGI cups were pre-coated with 15–30 µL of silicone oil per cup. Each size-3 hard gelatine PEG capsule (Qualicaps, Osaka, Japan) was filled with 20 mg of powdered sample. Aerosolization of the encapsulated powder was achieved using the Foradil Aerolizer (Novartis Pharmaceuticals UK Ltd., London, UK). Before beginning aerosolization, the air flow rate was verified and adjusted to 100 L/min using a Copley DFM 2000 flow meter in conjunction with an electronic digital flow meter (Copley, model DFM2). The liberated powder from each capsule was drawn through the NGI at a flow rate of 100 L/min for 2.4 s. At this flow rate, the cut-off diameters are 5.27, 2.40 and 1.32 mm at stages 3, 4 and 5, respectively [20]. After actuation, the leftover powder retained in both the capsule shell and aerolizer, as well as the powder deposited in the induction port and on each stage, were collected by rinsing thoroughly with a diluent (20:80% *v*/*v* water: methanol). The collected samples were then made up to a final volume of 100 mL (aerolizer and capsule shell, mouthpiece and induction port), 25 mL (stage 1 and micro-orifice collector, MOC) and 20 mL (stage 2–7). The amount of drug in each was quantified by HPLC.

Aerodynamic parameters, including emitted dose (ED) and fine particle fraction (FPF), were calculated as reported by Eedara et al. (2016) [23]. ED represents the total mass of the drug emitted from the aerolizer, determined by subtracting the leftover drug retained in the aerolizer and capsule shell from the total recovered dose (RD%). To assess FPF (%), which denotes the percentage of emitted dose capable of penetrating all the way to the deep lungs (aerodynamic diameter ≤ 5 µm), the dose deposited on stage 2 through to the MOC was measured relative to the emitted dose. The mass median aerodynamic diameter (MMAD) refers to the median aerodynamic diameter of the recovered particles, below which 50% of the particle population lies. Additionally, the geometric standard deviation (GSD) was utilized as a measure of the distribution of aerodynamic particle sizes. Both MMAD and GSD were determined using Copley Inhaler Testing Data Analysis software (CITDAS 3.10), with calculated values corrected for actual drug content per capsule.

### 2.12. HPLC Analysis

The HPLC procedures were fully validated prior to their routine use [24]. A Shimadzu (Kyoto, Japan) HPLC system consisted of an SPD-M20A photodiode array detector, an LC-20AD solvent delivery unit, a DGU-20A5 degasser and a SIL-20AC auto-sampler with Class-VP 7.4SP4 software. Chromatographic separations were performed using a reverse phase octadecyl (C_18_) column (150 mm × 4.6 mm, 4 µm, Phenomenex, Torrance, CA, USA) preceded by a C_18_ security guard column (4.0 mm × 3.0 mm, Phenomenex, Torrance, CA, USA). The samples (20 µL) were analysed in the isocratic mode using the following analytical conditions. For analysis, the mobile phase was composed of 30 mM orthophosphoric acid (pH 2.5 adjusted with triethylamine) and methanol (55:45% *v*/*v*), and the flow rate was 1 mL/min. The detection wavelengths for PYR, MOX and PA were 269 nm, 296 nm and 330 nm, while retention times were 3.5 min, 7.8 min and 17.7 min, respectively. The method was validated prior to use, and the accuracy and precision were within the specified limits of ±15% and <15%, respectively.

### 2.13. In Vitro Dissolution Study of Spray-Dried Powder Particles

The in vitro dissolution study of PYR-PA-LEU and PYR-MOX-LEU powder samples was carried out using a custom-made dissolution apparatus, as reported previously [25,26]. The dissolution apparatus is designed to mimic the air–blood interface and utilizes a small volume of dissolution medium mimicking the conditions of the respiratory tract. The custom-made apparatus consisted of a flow perfusion cell maintained at a temperature of 37 °C and utilized a dialysis membrane soaked in phosphate-buffered saline (PBS pH 7.3) as the air–blood barrier simulant (12,400 Da, Sigma Aldrich, St. Louis, MI, USA). The perfusate (PBS pH 7.3) was also maintained at a temperature of 37 °C with the help of a heated water jacket around the 100DM syringe pump (Teledyne ISCO, Lincoln, NE, USA), which pumped the perfusate at a flow rate of 0.4 mL/min, which then flowed beneath the dialysis membrane. The respirable fractions of the powder formulations were collected on glass coverslips using a modified Twin Stage Impinger, as reported previously [25,26], which were then placed above the dialysis membrane. The dissolution of powder particles took place above the membrane, which had 15 µL of dissolution medium (containing 1.5% *w*/*v* polyethylene oxide 5000 kDa and 0.4% *w*/*v* Curosurf^®^ in PBS) spread uniformly on it. The dissolved fractions of the drug which permeated through the dialysis membrane were collected via an outlet at the predetermined time points of 2, 4, 6, 8, 10, 15, 20, 25, 30, 45, 60, 90, 120, 180 and 240 min and the samples were analysed by HPLC for quantification of the drugs.

### 2.14. Stability Study

All spray-dried powder formulations were stored in airtight screw-capped glass vials at 30 ± 2% relative humidity under a desiccator at room temperature and were protected from light. The powder samples were tested at 0 and 3 months for the drug content, in vitro aerosolization, surface morphology and solid-state properties following the same methods mentioned above. 

### 2.15. Statistical Analysis 

All data were processed and represented as mean ± standard deviation values. Statistical analysis for comparison of groups was carried out by one-way analysis of variance (ANOVA) followed by a Student–Newman–Keuls post hoc test using GraphPad Prism 5 software (GraphPad Software, New York, NY, USA). *p* values less than 0.05 were considered statistically significant.

## 3. Results and Discussions

### 3.1. Preparation of Spray-Dried Powder Formulations and Estimation of Percentage Yield and Drug Content

The percentage yield for each formulation was calculated, and the results were summarized in Table 1. The percentage yield gives an estimation of the efficiency and reproducibility of the preparation process for each formulation. This is important, especially during scale-up and large-scale manufacturing, where a high process yield is ideal. LEU is normally used in inhalable formulations as an excipient to increase the yield as well as the aerosolization efficiency by enhancing the dispersibility of the powders [27,28]. The drug content of the SD powders estimated from HPLC analysis (Table 1) was within 90–100% of the theoretical drug content, indicating the chemical stability of drugs at spray-dried conditions.

### 3.2. Surface Morphology by SEM

**Figure 2:**
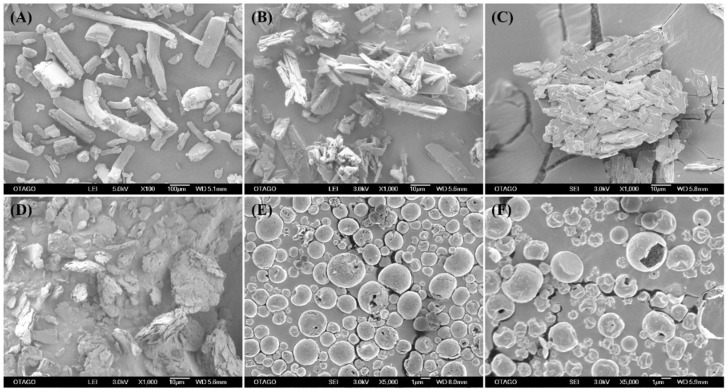
Representative scanning electron microscopic (SEM) images of supplied materials: (**A**) PYR, (**B**) MOX and (**C**) PA; (**D**) LEU; and spray-dried powder formulations: (**E**) PYR-PA-LEU and (**F**) PYR-MOX-LEU.

SEM was used to visualize the particle shape and size, which are the determinants of the aerosolization performance of dry powder formulations. The SEM images of the supplied materials and the spray-dried powder formulations are represented in Figure 2. The supplied raw materials had geometric sizes bigger than 10 μm with a varying range of size distributions. Specifically, the supplied PYR (Figure 2A) exhibited rod-shaped particles with a size of > 100 µm, indicating a crystalline form, which was confirmed later by the XRPD results. MOX (Figure 2B), in its supplied form, appeared as agglomerates of many long, rod-shaped crystals in various sizes. SEM of supplied PA (Figure 2C) showed irregularly shaped crystals composed of numerous thin flakes aggregating together. The excipient LEU (Figure 2D) appeared as large irregular particles in its supplied form when viewed under the SEM. 

The spray-dried powder formulations PYR-PA-LEU and PYR-MOX-LEU (Figure 2E,F) appeared as hollow, small, spherical particles with dimples. There were no visible crystals observed in the SEM images of spray-dried powders. The observation from SEM images suggested that the particle morphology was largely influenced by LEU. This can be explained by the surfactant-like properties of LEU, which facilitated its accumulation at an air–water interface. Moreover, the lower molecular weight of LEU, compared to the other components in the powder formulation, might have helped it competitively concentrate on the surface of the droplets and the resultant powder particle. This is based on the assumption that during the spray-drying process, the LEU surface layer collapsed, resulting from the rapid and complete evaporation of the solvent from the droplet, resulting in the dimpled porous structure, as observed in the SEM images [29]. In addition, the dimpled, hollow particles are likely to reduce the tapped density, minimize contacts between particles, and facilitate better dispersion and aerosolization by minimizing interparticle cohesive forces [30]. Similar to the previous reports in the literature [31,32,33], the effect of LEU in the present study corresponded to increased de-agglomeration and, thus, higher FPF values in those formulations spray-dried with LEU, as discussed below in Section 3.9.

### 3.3. Particle Size of Spray-Dried Powder Formulations

Particle size analysis was carried out for the spray-dried powder formulations (PYR-PA-LEU and PYR-MOX-LEU) using the SEM images. As shown in Table 1, both spray-dried powder formulations exhibited an average geometric diameter of less than 5 μm with a size range of 0.6–5.3 μm, indicating that they were within a suitable particle size range to avoid deposition in the oropharyngeal cavity by inertial impaction. 

### 3.4. Differential Scanning Calorimetry (DSC)

**Figure 3:**
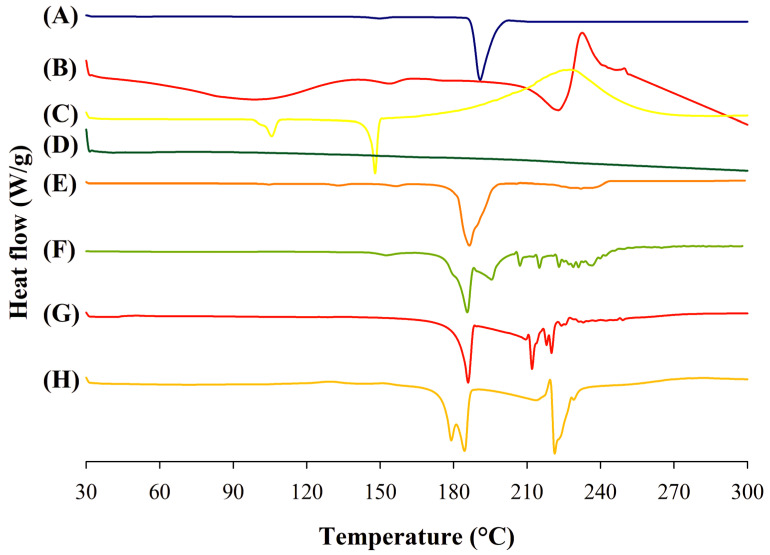
Differential scanning calorimetry (DSC) thermograms of supplied materials: (A) PYR, (B) MOX and (C) PA; (D) LEU; physical mixtures: (E) PYR-PA-LEU and (F) PYR-MOX-LEU; and spray-dried powder formulations: (G) PYR-PA-LEU and (H) PYR-MOX-LEU.

The thermal characteristics of the supplied materials and the spray-dried powder formulations were studied using DSC, and the thermograms are shown in Figure 3. In the case of supplied PYR, a phase transition peak at 150 °C was observed, indicating a polymorphic change of PYR at that temperature point (Figure 3A), and this transformation represents the α-polymorphic crystalline form of the supplied drug [34]. An endothermic peak at 190 °C showed that PYR underwent melting at that temperature. The DSC thermogram of supplied MOX (Figure 3B) showed a broad endothermic peak in the temperature range of 30–120 °C resulting from moisture loss, together with a solid phase transition peak at 150 °C and a broad melting endothermic peak at 223 °C. The DSC thermogram of supplied PA is shown in Figure 3C, which features a solid phase transition endothermic peak, a melting endothermic peak and a broad exothermic degradation peak at 105 °C, 148 °C and 227 °C, respectively. There were no obvious thermal events observed in the thermogram of supplied LEU over the temperature range of 30–300 °C (Figure 3D).

DSC thermogram of PYR-PA-LEU physical mixture (Figure 3E) showed solid phase transition and melting endothermic peaks of PYR and PA at 105 °C, 135 °C, 155 °C and 186 °C with a slight shift in peak positions. The presence of melting endotherms of PYR and PA indicates the crystalline nature of both drugs in their physical mixture without any polymorphic transformation. A slight shift in endothermic peak positions represents the degree of drug miscibility during melting. Similarly, the DSC thermogram of PYR-MOX-LEU physical mixture (Figure 3F) showed the solid phase transition and melting endotherm of PYR at the position similar to supplied PYR followed by a melting endothermic peak of moxifloxacin at ~200 °C indicating dissolution of moxifloxacin in molten PYR. To further confirm the physicochemical interaction between the drugs and LEU in physical mixtures, an XRPD analysis was conducted, the results of which are shown in Section 3.7. 

The spray-dried powder formulation, PYR-PA-LEU (Figure 3G), exhibited an endothermic melting peak of pyrazinamide at 186 °C and multiple endothermic peaks above 210 °C possibly due to the degradation of the components, which was confirmed from the observation of the charred residue left in the DSC pan at the end of the experiment. Similarly, PYR-MOX-LEU (Figure 3H) displayed a split endothermic peak at around 180 °C, which represented the melting of the crystalline pyrazinamide, and an exothermic peak followed by an instantaneous endothermic event at 221 °C to indicate recrystallization of amorphous moxifloxacin HCl and its following melting. The thermal events of these two formulations around 190 °C indicated the presence of the γ-polymorphic pyrazinamide in the respective formulations [34].

### 3.5. Thermogravimetric Analysis (TGA)

**Figure 4:**
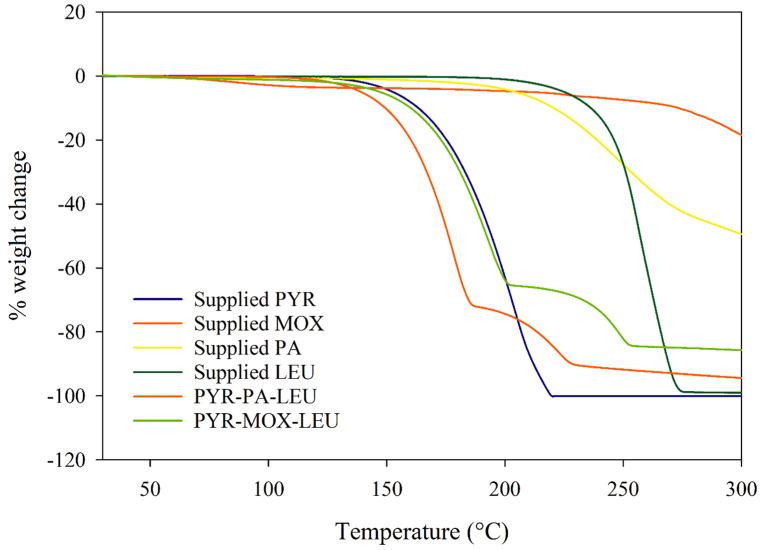
Thermogravimetric analysis (TGA) curves of supplied materials and spray-dried powder formulations.

The TGA curves shown in Figure 4 demonstrate the weight loss of each powder formulation as temperature increased from 30 to 300 °C. The supplied PYR exhibited a continuous weight loss starting at 150 °C, and rapid loss was observed after its melting point, i.e., 190 °C, which indicated the evaporation of the molten drug and complete 100% loss was observed before 210 °C. The weight loss of MOX started at 50 °C and reached a plateau state (~3.3%) after 110 °C, corresponding to water evaporation. As the temperature increased up to around 240 °C, the weight loss was minimal (~6.7%). Even at 300 °C, about 18.5% of the weight was lost, indicating melt degradation (charring) of the drug without any evaporation. Obvious weight loss of supplied PA started from 100 °C, and the rate increased from 200 °C. By the end of TGA, almost half of the total weight of PA was lost. Supplied LEU had a minor loss of weight up to the temperature of 250 °C, where a rapid weight loss was observed, and almost all the materials were lost after 275 °C. 

The PYR-PA-LEU formulation showed a weight loss from 100 °C corresponding to the loss of PA. From 150 °C, the rapid rate of weight loss is attributed to both PA and PYR. From 250 °C, the weight of LEU started to decrease. At the end of the experiment, almost 95% of the total weight was lost, and the remaining was the weight of half of the PA amount. Similarly, in PYR-MOX-LEU, the rapid rate of weight loss was due to PYR and MOX. However, the rate was slower compared to that in PYR-PA-LEU due to a slower loss of MOX. The remaining weight was also higher because of a higher amount of MOX in the formulation.

### 3.6. Hot-Stage Microscopy

**Figure 5:**
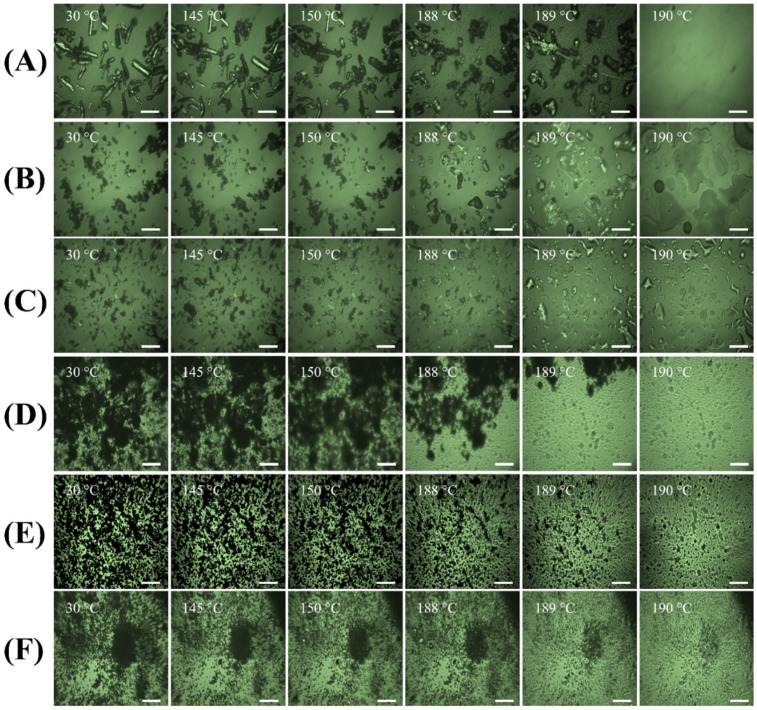
Representative hot-stage microscopy images (scale bars represent 100 µm) of supplied materials: (**A**) PYR, (**B**) MOX and (**C**) PA; (**D**) LEU; and spray-dried powder formulations: (**E**) PYR-PA-LEU and (**F**) PYR-MOX-LEU.

The hot-stage microscopy was used to visualize the change in powders with respect to the increase in temperature ranging from 30 to 300 °C. Supplied PYR (Figure 5A) exhibited as rod-shaped crystals with shining birefringence, and its solid phase transition occurred at around 150 °C where the crystals appeared darker, and less birefringence was observed. Then, it started to melt at 189 °C, and by 190 °C the melting was complete. This observation corresponded to the results of DSC and TGA. Melting of supplied MOX (Figure 5B) was observed to begin at 220 °C, followed by degradation after its melting point. Under the polarized microscope, supplied PA (Figure 5C) appeared as flake-shaped crystals with bright birefringence. PA had its phase transition at around 110 °C, showing more dark matter and started melting after 140 °C. Supplied LEU (Figure 5D) was shown to degrade at around 230 °C, which corresponds with the results from DSC and TGA. 

The spray-dried powder formulations appeared to be dark, fine powders, indicating their amorphous nature after the spray-drying process. Particles of PYR-PA-LEU powder (Figure 5E) started to lose mass at 170 °C and melted afterwards. In the particles from the PYR-MOX-LEU formulation (Figure 5F), the mass was visible from around 150 °C. At around 173 °C, there were some spots of shining birefringence, as observed in the figures, which might be due to the crystalline PYR polymorph.

### 3.7. X-ray Powder Diffraction (XRPD) Analysis

XRPD analysis was conducted for powder samples to study the crystallinity of each sample and its polymorphic form before and after spray drying. XRPD is important to understand whether the powder samples are crystalline or amorphous. Amorphous particles are thermodynamically unstable and tend to recrystallize, and changes in such properties ultimately lead to unexpected effects on the aerosolization performance of the powder formulations.

**Figure 6:**
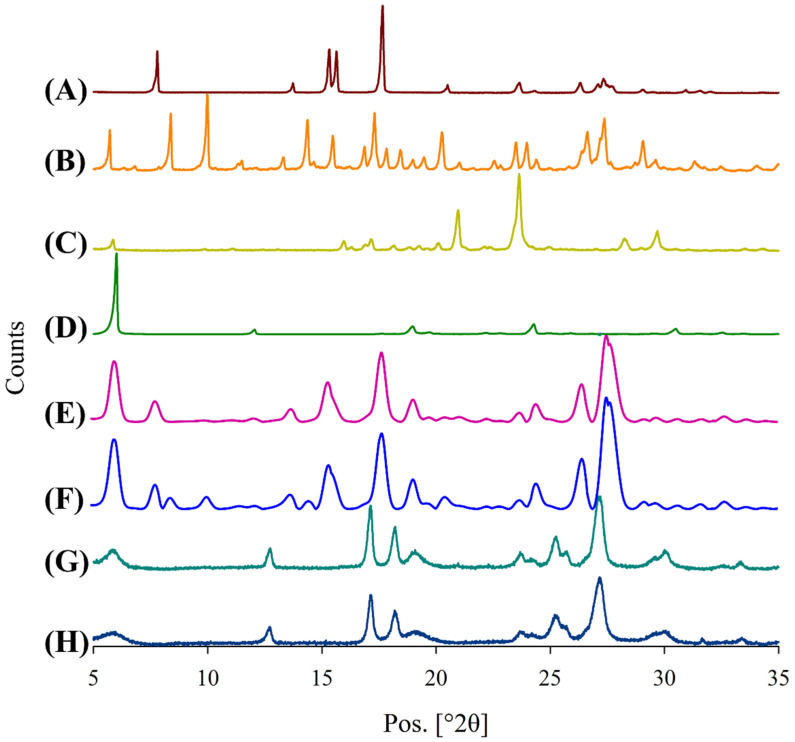
X-ray powder diffractograms (XRPD) of supplied materials: (A) PYR, (B) MOX and (C) PA; (D) LEU; physical mixtures: (E) PYR-PA-LEU and (F) PYR-MOX-LEU; and spray-dried powder formulations: (G) PYR-PA-LEU and (H) PYR-MOX-LEU.

Figure 6 shows the XRPD patterns of the supplied materials and the spray-dried powder formulations. The diffractogram of supplied PYR (Figure 6A) showed sharp diffraction peaks, suggesting that it had a predominant crystalline composition. The diffraction pattern is comparable to that of the α-polymorph of PYR reported previously [34,35]. The diffractogram of the supplied MOX (Figure 6B) also had multiple sharp diffraction peaks, suggesting its crystalline nature. Similarly, in the case of supplied PA and LEU (Figure 6C and Figure 6D, respectively), the diffractograms exhibited sharp diffraction peaks, suggesting they were also present in crystalline forms before the spray-drying process. The physical mixtures of PYR-PA-LEU (Figure 6E) and PYR-MOX-LEU (Figure 6F) produced a diffraction pattern that exactly matched with the superimposed diffractograms of respective combination of supplied materials, indicating the crystalline nature of drugs and LEU. The absence of new peaks in the diffractograms of physical mixtures ruled out the physicochemical interaction in their physical mixture.

The spray-dried powder formulations of PYR-PA-LEU and PYR-MOX-LEU (Figure 6G,H) showed similar patterns of diffractogram with peaks at positions 17.1°, 18.2°, 25.3°, 27.0° and 27.2° (2θ). These peaks corresponded to the peaks of γ-polymorph of PYR [35]. The fact that no other sharp peaks were present and the observation of a halo in the two diffractograms indicated the likelihood of MOX and PA being transformed into an amorphous form after spray drying. On the other hand, LEU still exhibited sharp peaks at position 6.0°, suggesting its crystalline nature even after the spray-drying process. Overall, we have observed that pyrazinamide went through polymorphic transformation from α-form to γ-form in spray-dried formulations. The presence of the γ-polymorphic form of PYR in both PYR-PA-LEU and PYR-MOX-LEU formulations produced identical diffractograms (Figure 6G,H). The mechanism and reason for the polymorphic change of PYR when it interacted with LEU remains unclear. Future studies need to investigate the mechanism of PYR polymorphic changes in these spray-dried combination powders. This is important because the polymorphism of PYR may significantly affect the inhalation formulation stability and aerosolization behaviour.

### 3.8. Attenuated Total Reflectance—Fourier-Transform Infrared (ATR-FTIR) Spectroscopy

**Figure 7:**
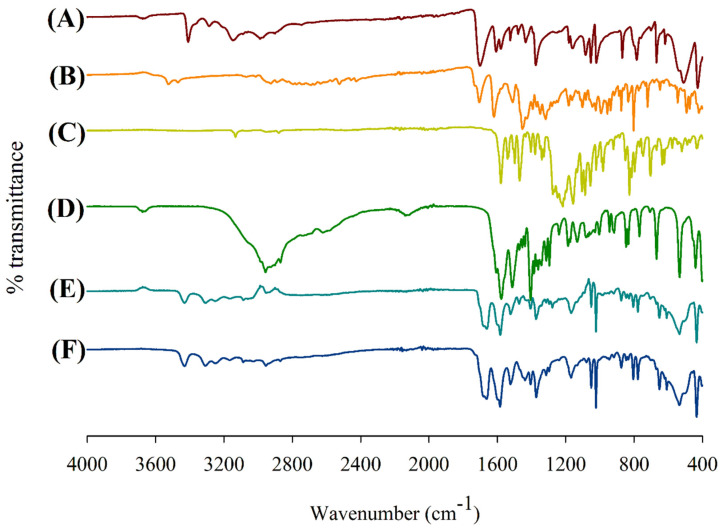
Attenuated total reflectance—Fourier-transform infrared (ATR-FTIR) spectra of supplied materials: (A) PYR, (B) MOX and (C) PA; (D) LEU; and spray-dried powder formulations: (E) PYR-PA-LEU and (F) PYR-MOX-LEU.

The ATR-FTIR spectra of the supplied materials and spray-dried powder formulations are presented in Figure 7. In the case of the supplied PYR, the N-H amide stretch is represented by the characteristic peaks at 3410 cm^−1^, 3287 cm^−1^ and 3146 cm^−1^ in the FTIR spectrum (Figure 7A). Similarly, the C=O and N-H amide stretch in the PYR structure are represented by peaks at 1702 cm^−1^ and 1607 cm^−1^; the ring vibrations are represented by peaks at 1580 cm^−1^, 1524 cm^−1^, 1479 cm^−1^ and 1434 cm^−1^; the ring C-N stretching is represented by peaks at 1375 cm^−1^, 1159 cm^−1^, 1085 cm^−1^, 1053 cm^−1^ and 1021 cm^−1^; and the α-polymorphic form is represented by the peaks for ring bending at 869 cm^−1^, 784 cm^−1^, 669 cm^−1^, 618 cm^−1^, 510 cm^−1^ and 424 cm^−1^. In the case of supplied MOX (Figure 7B), the O-H water, carboxylic acid stretch is represented by peaks at 3525 cm^−1^, 3470 cm^−1^, 2924 cm^−1^ and 2890 cm^−1^; the O-H carboxylic acid stretch corresponds to the peaks at 2924 cm^−1^, 2890 cm^−1^, 2525 cm^−1^, 2453 cm^−1^ and 2425 cm^−1^; the C=O cyclic ketone stretch is represented by peaks at 1705 cm^−1^ and 1602 cm^−1^; the C=C aromatic stretch and O-H carboxylic acid bending is represented by peaks at 1510 cm^−1^, 1456 cm^−1^, 1393 cm^−1^, 1371 cm^−1^, 1350 cm^−1^ and 1318 cm^−1^; the C-O phenol stretch is represented by peaks at 1183 cm^−1^, 1103 cm^−1^ and 1045 cm^−1^; and the ring vibrations indicating the presence of the drug in the hydrate crystalline form are represented by peaks at 993 cm^−1^, 956 cm^−1^, 937 cm^−1^, 875 cm^−1^, 834 cm^−1^ and 721 cm^−1^. The FTIR spectrum for supplied PA-824 (Figure 7C) showed characteristic aromatic C=C-H stretch peaks at 1579 cm^−1^, 3133 cm^−1^ and NO_2_ stretching peaks at 1470 cm^−1^, 1498 cm^−1^, 1540 cm^−1^, suggesting a structure corresponding to a nitro compound. The spray-dried powder formulations (Figure 7E,F) showed similar FTIR spectra with characteristic peaks at similar positions compared to the spectra of their supplied form; however, with decreased intensity, suggesting there was no chemical incompatibility or interactions between the drugs and the excipients.

### 3.9. In Vitro Aerosol Dispersion Performance

**Figure 8:**
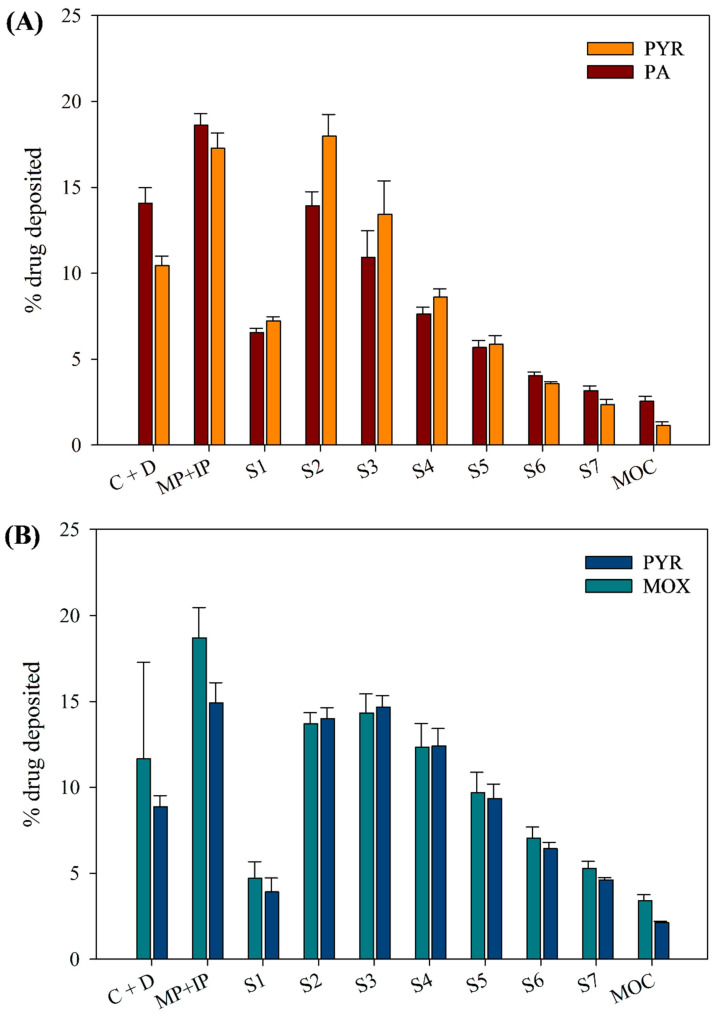
In vitro aerosol dispersion performance of spray-dried powder formulations, (**A**) PYR-PA-LEU and (**B**) PYR-MOX-LEU, using Next Generation Impactor™ (NGI™) at a flow rate of 100 L/min with an aerolizer device. Data represent mean ± SD (n = 3). C + D—capsule shell and device; MP + IP—mouthpiece and induction port; S1–S7—NGI stages 1 to 7; and MOC—micro-orifice collector.

The in vitro aerosolization performance of both PYR-PA-LEU and PYR-MOX-LEU formulations was similar when analysed using NGI (Table 2 and Figure 8). Both powder formulations had an ED of more than 83% and an FPF greater than 65%, suggesting good aerosolization behaviour with the potential to be delivered into the deep lungs. The MMAD values were also smaller than 5 μm for both formulations, further suggesting that the powder particles were of inhalable size. Between the two formulations, PYR-MOX-LEU powder showed higher ED and FPF than PYR-PA-LEU powder (*p* < 0.05), which is due to the smaller aerodynamic diameter of the PYR-MOX-LEU powder formulation as indicated by its MMAD values. Thus, smaller powder particles are more likely to be delivered into the deep lungs. 

### 3.10. In Vitro Dissolution Study of Spray-Dried Powder Particles

**Figure 9:**
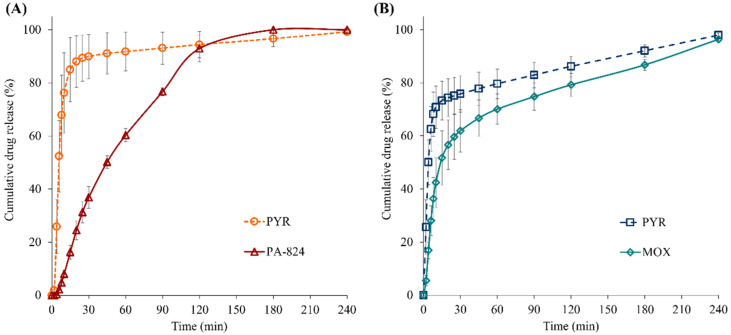
Permeation profiles of PYR, PA and MOX from respirable particles of spray-dried formulations (**A**) PYR-PA-LEU and (**B**) PYR-MOX-LEU in 15 µL of mucus simulant (1.5% *w*/*v* PEO in PBS) with lung surfactant; LS (0.4% *w*/*v* Curosurf) at 0.4 mL min^−1^ perfusate flow rate (PBS, pH 7.3). Data are means ± SD (n = 3).

Figure 9 shows the permeation (i.e., drug diffusion through the membrane into the perfusate medium) profiles of the PYR, PA and MOX from respirable particles of two spray-dried formulations (PYR-PA-LEU and PYR-MOX-LEU) in 15 µL of mucus simulant (1.5% *w*/*v* PEO in PBS) with lung surfactant, LS (0.4% *w*/*v* Curosurf), at 0.4 mL min^−1^ perfusate flow rate (PBS, pH 7.3). Both spray-dried formulations (PYR-PA-LEU and PYR-MOX-LEU) showed faster permeation of PYR with >75% PYR permeation in <30 min. This indicates rapid dissolution of water-soluble, low molecular weight (123.1 g/mol) PYR from the respirable composite dry powder particles compared to the less soluble, high molecular weight PA (359.2 g/mol) and MOX (401.4 g/mol). PYR, from a spray-dried large porous particle powder formulation, is reported to be rapidly absorbed into the systemic circulation after intra-tracheal insufflation in laboratory rats with an average t_max_ (time to maximum plasma concentration) of 11.4 min [36]. These observations from both in vitro and in vivo studies suggest that PYR, from a spray-dried powder formulation, is likely to undergo rapid dissolution in the lungs and be readily absorbed into the systemic circulation. The permeation profile of hydrophobic PA from spray-dried formulation PYR-PA-LEU was slower compared to that of PYR and linear over 120 min with >80% drug permeation, indicating slow dissolution of PA from composite particles. The pattern of dissolution followed by permeation for MOX from PYR-MOX-LEU formulation (Figure 9B) is similar to that from MOX respirable particles from a spray-dried formulation [25].

### 3.11. Stability Study

#### 3.11.1. Drug Content and In Vitro Aerosol Dispersion Performance upon Storage

**Figure 10:**
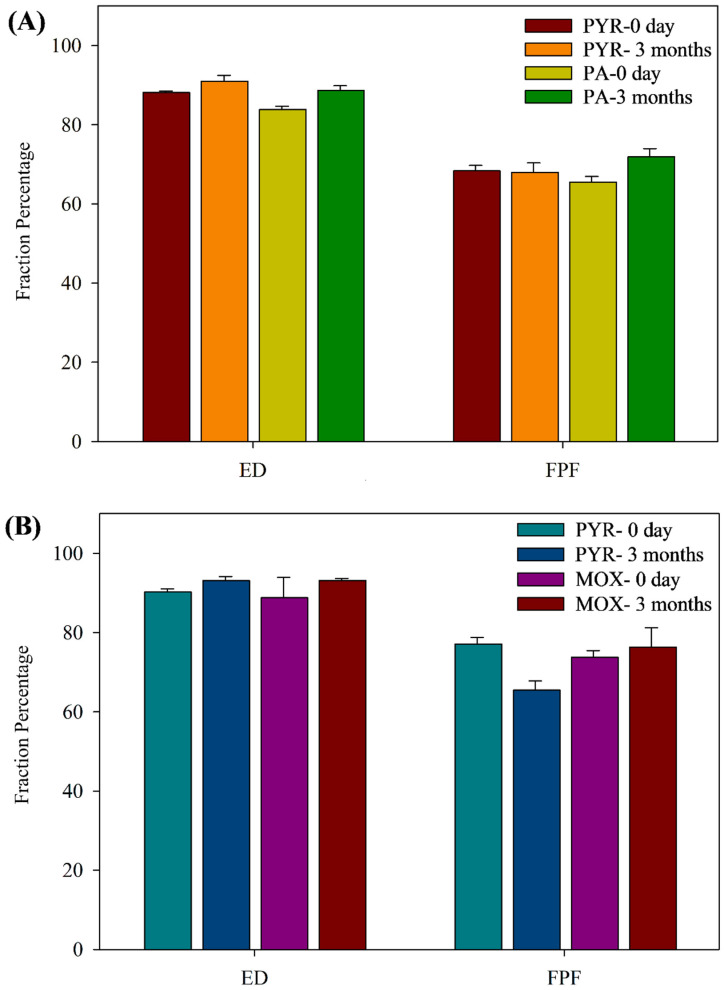
Comparison of aerodynamic parameters for spray-dried powder formulations (**A**) PYR-PA-LEU and (**B**) PYR-MOX-LEU at day 0 and at 3 months using Next Generation Impactor™ (NGI™) at a flow rate of 100 L/min with an aerolizer device. Data represent mean ± SD (n = 3). ED—emitted dose; FPF—fine particle fraction.

The drug content of both formulations was within 90–100%, like fresh samples, which indicates the chemical stability of prepared spray-dried formulations for the short term. Figure 10 compares the aerodynamic parameters of each drug in the two spray-dried powder formulations (PYR-PA-LEU and PYR-MOX-LEU) at day 0 and at 3 months. In the PYR-PA-LEU (Figure 10A), both PA and PYR remained similar in terms of ED and FPF, with the values for PA increased slightly at 3 months. In the PYR-MOX-LEU formulation (Figure 10B), PYR had similar ED at day 0 and at 3 months; however, the FPF decreased remarkably at 3 months (*p* < 0.05). MOX had similar aerodynamic parameters, which indicate its aerosolization stability over 3 months. Upon comparison of these aerosolization stability results, we can observe that PA might have helped protect PYR from degradation. However, this is contrary to the results from SEM analysis of stored samples (in the following discussion), which showed the formation of solid bridging between particles. Further study is necessary to investigate the mechanism behind this observation.

#### 3.11.2. Morphological Changes during Storage

**Figure 11:**
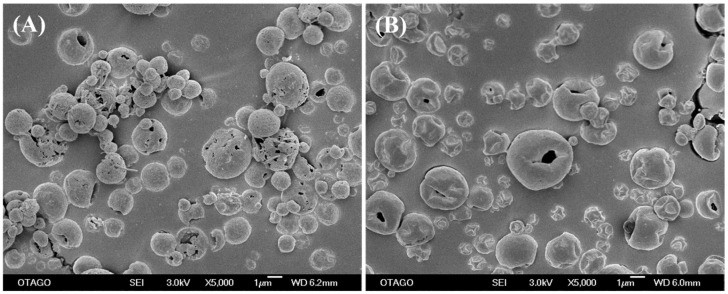
Representative scanning electron microscopic (SEM) images of spray-dried powder formulations (**A**) PYR-PA-LEU and (**B**) PYR-MOX-LEU observed at 3 months.

Figure 11 represents the changes that occurred over three months of storage of the spray-dried powder formulations viewed under SEM at 5000× magnification. PYR-PA-LEU powder particles (Figure 11A) showed the formation of solid bridging between particles at three months, suggesting morphological changes in the particles. As the aerosolization parameter values (for ED and FPF) remained high for this formulation, the solid bridging might not be strong enough to keep particles aggregating together. In contrast, PYR-MOX-LEU (Figure 11B) did not exhibit any difference in their morphology at 3 months compared to the initial morphology of freshly prepared samples. Although some variation in aerosolization parameters of stored samples of PYR was observed from NGI analysis, no features of instability could be observed in the SEM images of the PYR-MOX-LEU powder samples. Therefore, further investigation regarding both physicochemical and aerosolization changes upon storage of these powder particles is necessary.

#### 3.11.3. Crystallinity Changes upon Storage

**Figure 12:**
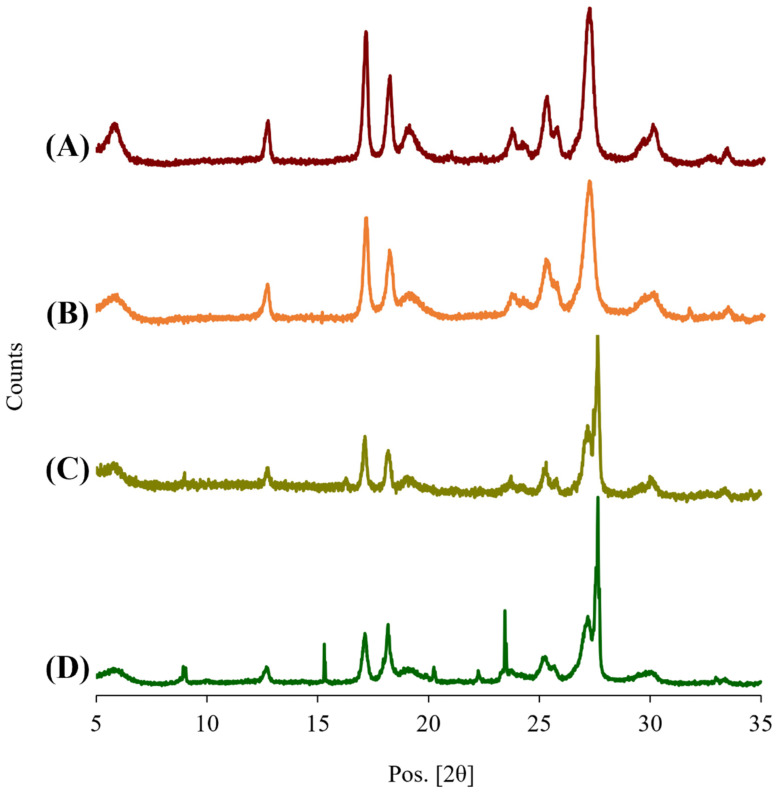
X-ray powder diffractograms (XRPD) of spray-dried powder formulations at 0 days (A, PYR-PA-LEU, and B, PYR-MOX-LEU) and 3 months (C, PYR-PA-LEU, and D, PYR-MOX-LEU) after storage for stability study.

Due to the variation in the stability of the formulations, an XRPD analysis was also performed, the results of which are shown in Figure 12. Although the diffractograms of spray-dried formulations stored for 3 months (Figure 12C,D) differed in peak intensity compared to that from the freshly prepared samples (Figure 12A,B), they shared similar peak positions. They all exhibited sharp peaks at 17.2°, 18.2°, 25.2°, 27.3° and 27.6° (2θ), indicating that they have similar crystallinity with PYR maintaining its gamma polymorphic form [35]. Although further investigations on the stability of these combination powders are required in future studies, an initial investigation in this study showed the good stability of PA and MOX in the combination formulations.

## 4. Conclusions

High-dose DPI formulations containing either PYR and PA-824 or PYR and MOX were developed in this study using a spray-drying technique. To ensure that high doses of drugs can be delivered to the deep lungs in order to target the macrophages containing the mycobacteria, L-leucine was used as an aerosolization enhancer. The powder particles spray-dried with L-leucine typically exhibited porous, spherical morphology with a corrugated surface and size less than 5 μm in diameter. The rough surface of the powders helped reduce the inter-particulate forces, improved powder dispersion and aerosolization and facilitated the delivery of the powders to the deep lungs. In addition, incorporation of L-leucine in the powder formulations resulted in a polymorphic change of pyrazinamide from α-form to γ-form. The stability study conducted for three months for the PYR-PA-LEU and PYR-MOX-LEU formulations suggested that PA-824 and MOX within those combinations showed good stability; however, pyrazinamide had a significantly lower FPF at 3 months. This observation suggested the need for further investigation to improve the stability of pyrazinamide in fixed-dose combination powders for inhalation.

## Figures and Tables

**Table 1:** Drug content, process yield and particle size (n ≥ 300 particles) of the spray-dried powder formulations (data are means ± standard deviations; n = 3 for drug content; for yields, data are from a single determination).

Parameters	PYR-PA-LEU		PYR-MOX-LEU	
	PYR	PA	PYR	MOX
Drug content (%)	68.31 ± 0.02	10.12 ± 0.01	57.07 ± 0.03	15.18 ± 0.01
Process yield (%)	46.0		60.2	
Mean particle size (µm)	1.9 ± 0.6		2.4 ± 0.7	
Particle size range (µm)	0.6–4.1		1.0–5.3	

PYR: pyrazinamide; PA: PA-824; MOX: moxifloxacin hydrochloride; LEU: L-leucine.

**Table 2:** The aerodynamic parameters for spray-dried powder formulations using Next Generation Impactor™ (NGI™) at a flow rate of 100 L/min with an aerolizer device. Data represent mean ± SD (n = 3).

Parameters	PYR-PA-LEU		PYR-MOX-LEU	
	PYR	PA	PYR	MOX
% Recovery	87.93 ± 3.41	87.1 4 ± 2.38	91.31 ± 1.13	100.87 ± 11.29
% Emitted Dose	88.12 ± 0.38	83.84 ± 0.86	90.29 ± 0.80	88.84 ± 5.09
% FPF	68.34 ± 1.39	65.55 ± 1.42	77.15 ± 1.65	73.80 ± 1.70
MMAD (µm)	2.90 ± 0.09	2.58 ± 0.11	2.09 ± 0.10	1.99 ± 0.11
GSD	1.90 ± 0.08	2.13 ± 0.12	1.84 ± 0.48	2.21 ± 0.03

PYR: pyrazinamide; PA: PA-824; MOX: moxifloxacin hydrochloride; LEU: L-leucine, FPF: fine particle fraction; MMAD: mass median aerodynamic diameter; GSD: geometric standard deviation.

## Data Availability

Not applicable.
